# Structure and hydrogen bonding at the limits of liquid water stability

**DOI:** 10.1038/s41598-017-18975-7

**Published:** 2018-01-29

**Authors:** Flaviu Cipcigan, Vlad Sokhan, Glenn Martyna, Jason Crain

**Affiliations:** 1 IBM Research UK, Hartree Centre, Daresbury, WA4 4AD United Kingdom; 2 STFC Daresbury Laboratory, Daresbury, WA4 4AD United Kingdom; 3 IBM T. J. Watson Research Center, Yorktown Heights, New York, 10598 USA

## Abstract

Liquid water exhibits unconventional behaviour across its wide range of stability – from its unusually high liquid-vapour critical point down to its melting point and below where it reaches a density maximum and exhibits negative thermal expansion allowing ice to float. Understanding the molecular underpinnings of these anomalies presents a challenge motivating the study of water for well over a century. Here we examine the molecular structure of liquid water across its range of stability, from mild supercooling to the negative pressure and high temperature regimes. We use a recently-developed, electronically-responsive model of water, constructed from gas-phase molecular properties and incorporating many-body, long-range interactions to all orders; as a result the model has been shown to have high transferability from ice to the supercritical regime. We report a link between the anomalous thermal expansion of water and the behaviour of its second coordination shell and an anomaly in hydrogen bonding, which persists throughout liquid water’s range of stability – from the high temperature limit of liquid water to its supercooled regime.

## Introduction

Water exhibits a remarkable set of anomalous properties even under familiar conditions near ambient temperature and pressure. These include its temperature of maximum density at ambient pressure, its high dielectric constant, high surface tension and negatively sloped melting curve^1^. Water also displays an unusually high critical temperature, terminating the liquid-gas coexistence curve. Yet more anomalies emerge near the limit of liquid water stability. At low temperatures and near ambient pressures, liquid water remains in a metastable state trapped below the freezing point in a *supercooled* state^2^. Here the existence of a density maximum (just above freezing) below which the thermal expansion is negative, a potential hidden critical point, and maxima in thermodynamic responses have been discussed in the literature^1^ but are difficult to study and quantify both experimentally and via computer modelling due to length and time scale constraints.

The standard picture postulates that water anomalies arise from a competition^3^ between two local structures: a low-density, ordered structure and a high-density, disordered one. The evidence for this picture comes from both experiment and simulation. Experimentally, water can be vitrified into three types of glasses^4^: low density amorphous (LDA), high density amorphous (HDA) and very high density amorphous (VHDA)^5^. These glasses should therefore represent free energy basins in liquid water. Indeed, the existence of water ‘polyamorphism’ inspired the hypothesis of a liquid-liquid transition^6^ in supercooled water, which is still vigorously debated^7–12^ as experiments are difficult to perform in deeply supercooled water due to the short freezing timescales and simulations are challenged by length and timescales required for adequate sampling.

Simulation provides a viable and comprehensive route to study water’s structure, with the caveat that the chosen model may not reproduce all the essential physics of water. The structure of simulated liquid water has been explored using various order parameters, collective variables that encode structural motifs in a set of unique numbers. The tetrahedrality parameter (Q)^13^ measures local structure in the first coordination shell. The local structure index (LSI)^14^ measures the distance between the first and second shell, relating with the order and disorder in the gap between the two coordination shells. The distance to the fifth neighbour (*d*_5_)^15^, the neighbour fluctuating between the first and second shell, has also been used with success in assessing the structural order of liquid water.

Numerous studies have coupled the order parameters described above to anomalies in liquid water. Intrinsic structures, obtained by quenching instantaneous frames of a molecular dynamics trajectory, have a bimodal distribution of the LSI^16^, with two peaks, each associated with low-density and high-density structures. Similarly, *d*_5_ can be used as a discriminator^17^ between low density and high density phases in a thermodynamic model of liquid water.

The connectivity of the hydrogen bonding network is another measure of structural order in liquid water. The classical picture is of molecules that are mostly four-coordinated, with two hydrogen donor bonds, starting from a hydrogen atom and ending on an oxygen atom, and two hydrogen acceptor bonds, starting from an oxygen and ending on a hydrogen. Recent research has challenged this picture, with X-ray adsorption spectroscopy and X-ray Raman scattering suggesting that only two strong hydrogen bonds per water molecule are consistent with measured spectra^18^. This result is currently interpreted as an asymmetry in contact strength between acceptor and donor bonds, observed in both electronic structure calculations^19,20^ and simulations of an electronically coarse-grained model of water^21^.

Here, we study the structure of water throughout its region of stability, from its supercooled and stretched state through to its high temperature, high pressure regime. We employ a computationally efficient quantum-classical QDO (Quantum Drude Oscillator) model for water^22–25^, in which a coarse-grained molecular electronic subsystem used to provide the complete set of long-range many-body electronic responses. The parameters of QDO-water are derived from single molecule responses and the potential energy surface of a dimer. Path integral sampling yields a Born–Oppenheimer surface for the nuclei in a non-perturbative way, and thereby generates long-range interaction terms to all orders. These terms include many-body polarization, dispersion interactions, and polarization–dispersion cross interactions beyond the dipole approximation. Thus, electronic responses are unfiltered by artificial truncation and no environmental bias or symmetry is imposed a priori, such as the case for models with fixed functional forms fit to condensed phase properties. As a result, QDO-water predicts a realistic liquid under ambient conditions, with structural features and thermodynamic properties in excellent agreement with experiment^22,26^. Moreover, both branches of the the liquid-vapour coexistence curve, the liquid-vapour critical point, the temperature of maximum density, surface tension, enthalpy of vaporisation, structure of ice II, and supercritical isotherms are in excellent agreement with experiment^21,24^, which is an unprecedented transferability for computer water.

Using QDO-water, we make contact with the order parameters measuring water’s structure and study the distribution of the nearest neighbours. In particular, we focus on the ambient asymmetry in the connectivity of water’s hydrogen bonding network to cover a wider range of conditions.

## Methods

### Simulation details

We performed a series of adiabatic path integral molecular dynamics simulations for quantum Drude oscillators (APIMD-QDO)^23^ in the canonical (*NVT*) ensemble for a system containing 300 water molecules in a cubic box periodically replicated in three dimensions. The classical nuclear subsystem of the QDO model for water^24^ was integrated with a timestep of 0.15 fs, and in the path integral discretisation we used Trotter number *P* = 96. Separate thermostats were used for nuclear and drudon degrees of freedom. The nuclear subsystem was driven to a desired temperature by a Nosé–Hoover chain^27^, whereas the drudons were kept at an order of magnitude higher temperature by a separate thermostat. Full set of model parameters including empirical short-range repulsion can be found elsewhere^24^. In addition to the electrostatic interactions which are handled by Ewald summation the model includes only exponentially decaying short-range repulsion and has therefore weak system-size dependence due to fluctuation-dependent properties. The systems were equilibrated for 100–300 ps and the statistics collected for further 300–1000 ps. Classical simulations using TIP4P/2005 model^28^ were performed using NAMD^29^ using the same system size and thermodynamic states as used in QDO water simulation.

### Model validation

**Figure 1:**
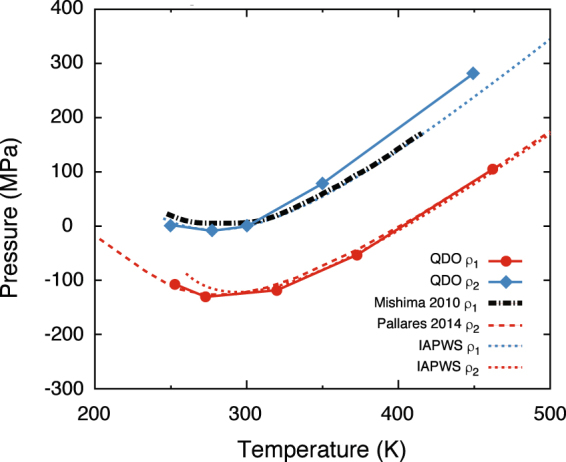
The pressure of 300 QDO-water molecules as a function of temperature at two constant volumes: *ρ*_1_ = 51.8002 mol/l (red) and *ρ*_2_ = 55.3173 mol/l (blue). The dashed line represent equivalent data digitized from Pallares *et al*.^32^ (*ρ*_1_, experimental estimates via simulations of TIP4P/2005). The dotted lines represent the corresponding isotherms of the IAPWS-95 reference equation of state for water^30^. The black dot-dashed line corresponds to an interpolated isochore based on the experimental data measured by Mishima^31^.

The first step is to validate the predictions of QDO-water in the supercooled and stretched regions. To do so we choose a series of experimental and computational benchmarks: (a) the IAPWS-95^30^ equation of state, which has been verified experimentally under strict thermodynamic conditions and mild supercooling; (b) the positive pressure experiments of Mishima^31^; (c) the negative pressure experiments of Pallares *et al.*^32^ and (d) simulations using a general-purpose model of water (TIP4P/2005) conducted by us and digitized from Singh *et al*.^17^. In order to perform the desired comparisons, the behaviour of QDO-water was sampled on two isochores: *ρ*_1_ = 51.8002 mol/l, matching the experiments of Pallares *et al.*^32^ and *ρ*_2_ = 55.3173 mol/l, close to ambient density, and on one isotherm: *T* = 230 K. Fig. 1 shows the pressure as a function of temperature compared with experimental and theoretical benchmarks.

The predictions of QDO-water match experiments on both isochores, showing that QDO-water’s validity extends to the supercooled and stretched liquid. Furthermore, by taking the minimum of pressure as a function of temperature on the *ρ*_2_ = 55.3173 mol/l isochore, a temperature of maximum density (TMD) is predicted at {*ρ* = 55.3173 mol/l, *T* = 271(2) K}, in good agreement with the experimental value of *T *= 277.13 K and our previous estimate of 278.6(20) K^24^ based on a direct sampling of the ambient pressure isobar. These results combined with previous work demonstrate that QDO-water describes well ambient water^26^, supercritical water^24^ the liquid-vapour interface^21^, ice II^26^ and now also supercooled and stretched water.

Convergence was checked by observing the time evolution of properties of the system. We note that the relaxation time of real water is shorter than a few nanoseconds in the region we sampled, which is shorter than our simulation time.

## Results

In this section we present our analysis of the structure of water. We begin with a study of the second coordination shell, followed by an analysis of the hydrogen bonding connectivity where we show that the asymmetry in hydrogen bonding persists over the whole range of temperatures and pressures studies. We link the structure of water at low and high density with that of amorphous ices and connect the behaviour of its dipole moment with two structural order parameters: the local structure index and tetrahedrality.

### Second coordination shell drives negative thermal expansion

Even at ambient temperature and mild supercooling, the structure of liquid water is a mixture of low density and high density structures. The competition between these structures is assumed to be responsible for two particular anomalies of liquid water: its density maximum and the resulting negative thermal expansion.

**Figure 2:**
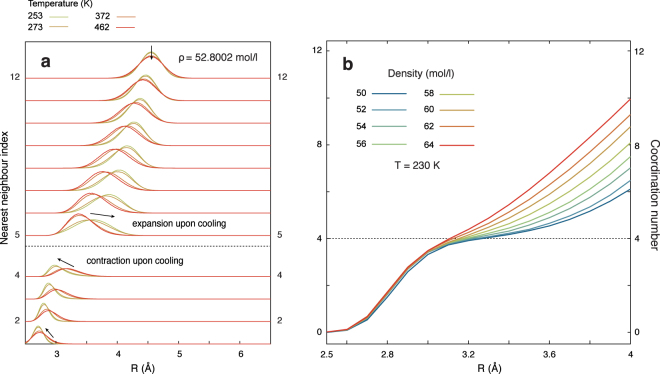
Structure of QDO-water upon cooling and supercooling. (**a**) Probability distribution of each neighbour (arbitrary units) as a function of temperature, showing the range of distances from a central molecule the neighbour occupies. (**b**) The coordination number as a function of density at *T* = 230 K.

The role of the second coordination shell in driving these anomalies is emphasised by the span of distances occupied by each neighbour, as shown in Fig. 2a. Upon cooling, the four nearest neighbours forming the first shell contract (i.e. normal behaviour). In contrast, the fourth to twelfth neighbours, part of the second shell, expand upon cooling (i.e. anomalous behaviour). Therefore, the second coordination shell acts to give water its negative thermal expansion. The density maximum and the return to positive thermal expansion then coincide with a cessation of the expansion of the second coordination shell with decreasing temperature; thus the free energy balance between local tensile stretching and increasing order is reached.

The role of the second coordination shell in distinguishing between low density and high density structures is further emphasized by examining the coordination number. Up to a value of 4 (i.e. full tetrahedral coordination), it changes weakly with density (see Fig. 2b). This means that the first coordination shell is highly incompressible, with its structure remaining relatively rigid across densities, reasonable behaviour for a strongly associating liquid.

### Hydrogen Bond Populations across liquid water’s stable range

**Figure 3:**
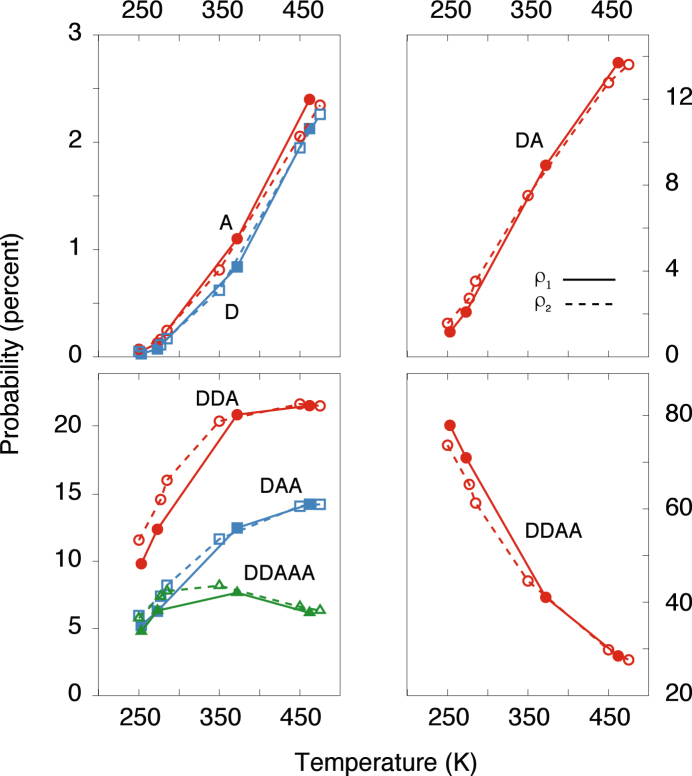
The probability of a given hydrogen bonding motif (assigned using the criterion in ref.^35^) as a function of temperature from simulations of QDO-water at two constant densities of *ρ*_1_ = 51.8002 mol/l (continuous lines) and *ρ*_2_ = 55.3173 mol/l (dashed lines).

The nature of the coordination shells of liquid water is a direct consequence of its propensity for hydrogen bonding. Fig. 3 shows the evolution of the probability of each hydrogen bonded motif as a function temperature on the two isochores. Each motif is labelled with the number of hydrogen acceptor and hydrogen donor bonds (for example, DA means “one donor and one acceptor bond”).

The hydrogen bonding structure behaves upon supercooling as expected for a strongly coordinating liquid. The tetrahedrally-coordinated motif increases upon cooling and supercooling while the three hydrogen-bonded motifs decreasing in population. What is interesting to notice is that, even at the lowest of temperatures, two anomalies are still present. First, the asymmetry between DDA and DAA, responsible for the surface orientation of water molecules^21^ survives even at the deepest of accessible supercooling. Second, the five-hydrogen bonded motifs are still present, their population showing little change between 250 K and 450 K.

At high temperatures, the story is different. Above *T* = 450 K (at sufficiently high density), water is still a liquid yet only 20% of the molecules are DDAA. Three hydrogen bonded motifs dominate, with around 15% DAA and 20% DAA. Upon cooling, the first shell becomes predominantly tetrahedral, with DDAA being the predominant motif.

### Structural relationship between supercooled water and amorphous ices

**Figure 4:**
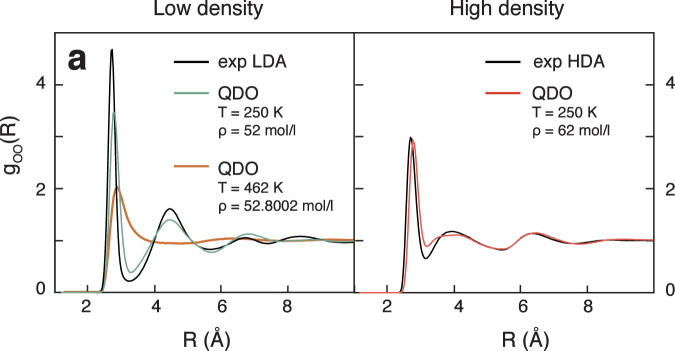
The radial distribution functions of QDO-water at *T* = 250 K and *ρ* = 52 and 62 mol/l compared with high and low density amorphous water measured by Finney *et al*.^33^.

The oxygen–oxygen radial distribution function at high and low density at *T* = 230 K (Fig. 4) reveal that QDO-water predicts two distinct local structures in supercooled water. These structures correspond closely to experimental measurements of high density amorphous (HDA, 64.76 mol/l) and low density amorphous (LDA, 51.86 mol/l) ices measured at *T* = 80 K^33^.

At low density, the structure is ice-like, with a well separated first and second coordination shell and little occupancy of “interstitial” sites between the two. At high density, the second shell moves to smaller radii with a decrease in the peak of the first shell to achieve a higher packing. These motifs are significantly different from the structure of QDO-water at high temperature, where only the first shell is strongly associated.

### Coupling between the electronic responses and structural order

The order parameters traditionally used to describe the anomalous behaviour of water and the mixture between two different structures at the local structure index (LSI)^14^ and tetrahedrality parameter (Q)^13^ (see Methods definitions). Here we study the relationship of these parameters with the behaviours of the first and second shell seen above.

The LSI measures the separation between the first and second coordination shells, with high values corresponding to a widely separated first and second shells. The parameter Q is a measure of first shell structural order. It takes a value of zero for a structureless shell and reaches one for perfect tetrahedral order. We also consider the molecular dipole moment, which we have shown to be a good descriptor of the subtle difference in local structure and hydrogen bonding patterns in polar fluids^26^.

**Figure 5:**
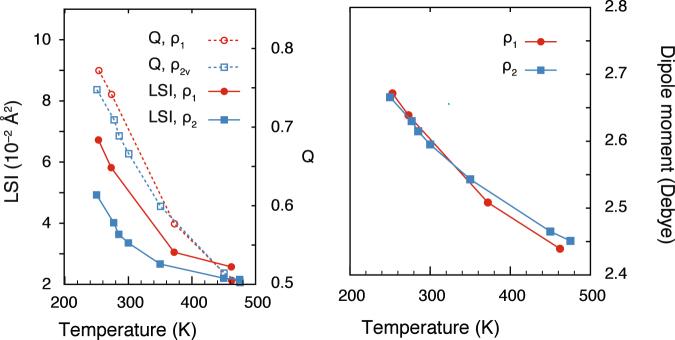
Order parameters as a function of temperature at a constant density of *ρ*_1_ = 51.8002 mol/l (red circles) and *ρ*_2_ = 55.3173 mol/l (blue squares).

Fig. 5 shows the variation of the LSI, Q and dipole moment as a function of temperature and density. Since the first shell structure changes little with density, we expect that Q and the dipole moment would show a weak density dependence while the LSI would show a strong density dependence. This is indeed observed, confirming the different compression mechanisms of the two shells and the importance of studying order parameters suitable for each length scale.

The dipole moment displays no anomaly, just a continuous increase upon cooling and supercooling, reflecting the increasing order in the first coordination shell. The dipole moment also reflects the incompressibility of the first coordination shell, showing little dependency on density.

## Discussion

We have explored the evolution of the structural and electronic responses of water across the full range of conditions over which a stable or metastable liquid form can be identified. We now seek to develop a picture of liquid water extending into the deeply supercooled and stretched regimes.

To develop better understanding of this challenging regime, we have performed a simulation study using QDO-water. This model allows a first direct prediction of the behaviour of the supercooled and stretched liquid based on a model derived entirely from properties of isolated molecules, thereby eliminating the parametrization bias typically introduced by fitting a physically limited force law to the properties of condensed phases at modeller-selected state points. QDO-water is electronically adaptive to changing environments since all many-body polarization, dispersion, and cross interactions are included nonperturbatively. As a result, QDO-water has been found to be predictive across the phase diagram^21–26^.

In our study of H-bonding in the supercooled regime, we have found that the distance to the *i*th neighbour is an insightful order parameter. It shows that while first coordination shell contracts upon cooling at a constant volume (an expected behaviour), the second shell expands (negative thermal expansion). Since water has positive thermal expansion above the temperature of maximum density and negative thermal expansion below, it means that the second shell behaviour is dominant at low temperatures.

The above observation is consistent with an increasingly tetrahedral liquid: when the first shell becomes predominantly four-coordinated, its density changes less with temperature, since this would imply a restructuring of the bonds. At high temperatures, the first shell has more degrees of freedom, since tetrahedral populations are less dominant. Thus, at high temperatures, we have a thermal expansion coefficient dominated by the first shell, leaving a positive thermal expansion. At low temperatures, when the first shell is mostly tetrahedral and dominated by strong hydrogen bonds, the second shell dominates, leading to negative thermal expansion. This observation is also consistent with studies of translational order in the first and second shell, which show that the anomaly in the translational order parameter is mainly caused by the behaviour of the second shell^34^.

We have also observed that the first shell is relatively incompressible at a constant temperature, with the density variations being caused by changes in coordination number in the second shell. This suggests two mechanisms of density change in water. The first is a shortening of nearest neighbour distances, which is visible in the behaviour of the first shell. The second is a collapse of oxygen–oxygen–oxygen angles between first shell and second shell molecules. This keeps tetrahedral cages rigid but packs them closer together. These two restructuring mechanisms balance thus allowing water’s peculiar anomalies to emerge from the molecular physics.

The hydrogen bonding asymmetry between DAA and DDA molecules persists throughout the temperature and pressure range studied. Thus, it is present in the supercooled limit, acting to frustrate freezing into mainly tetrahedral motifs. It is also present at high temperatures, where three-hydrogen bonded molecules form the dominant motifs. The asymmetry is balanced by the appearance of five-hydrogen bonded species, DDAAA, whose number is an indicator of the strength of the asymmetry (the more molecules are DDAAA, the more the asymmetry between DAA and DDA). The population of these motifs stays fairly constant, with a weak dependence on both temperature and density. This shows that, while the hydrogen bonding asymmetry is present across the range of stability of liquid water, it is only weakly affected by the state point.

At high temperatures, the molecular dipole moment, a sensitive reporter of the local environment^26^, is intermediate between its low isolated gas phase value (*μ *= 1.85 Debye) and the value found in the ambient liquid (*μ *= 2.6 Debye). The dipole moment is enhanced by almost 15% on cooling from the high- to low-temperature limits of the liquid. Throughout this range the predicted isotherms are in excellent agreement with experimental datasets including those that extend near the tensile stress limit of the liquid.

As evidenced by the partial radial distribution functions, the low- and high-density regions of the supercooled liquid bear a similarity to the local environments observed in high and low density glassy ices via neutron diffraction measurements at *T *= 80 K. QDO-water therefore points to a close correspondence between the compressed microstates in the supercooled liquid and amorphous solids.

In summary, we have used QDO-water to reveal two new structural insights into the hydrogen bonding structure of liquid water. The first and second coordination shells of liquid water show opposite behaviour upon cooling, with the first contracting and the second expanding. In the low temperature limit, highly-coordinated, four-hydrogen bonded motifs dominate and thus a first shell that behaves like a solid upon compression and thermal expansion. This physics leads to the emergence of water’s anomalies – negative thermal expansion, with the second shell dominating its thermal properties. At the high temperature limit, the picture is different, with the first shell being formed of lower coordinated motifs that are easier to restructure upon compression, heating, or cooling. The asymmetry between DDA and DAA hydrogen bonding motifs persists at all conditions, with DDAAA species having a relatively constant probability across water’s limits of stability. Lastly, these findings alongside good match to experimental benchmarks have extended the validity of QDO-water throughout the region of stability of liquid water, from its supercooled regime through to its stretched and high temperature limits; it also importantly allows us to reliably reveal the essential physics of hydrogen bonding over an extraordinary range of temperatures and pressures.
